# Rare Gastric Metastasis From Cervical Cancer: A Case Report

**DOI:** 10.1002/ccr3.70181

**Published:** 2025-02-05

**Authors:** Fariha Hasan, Yumna Timsaal, Tanay‐Veer Gandhi, Hadi Shojaei, Michael Schwartz, Adib Chaaya

**Affiliations:** ^1^ Department of Internal Medicine Cooper University Hospital Camden New Jersey USA; ^2^ Dow University of Health Sciences Karachi Pakistan; ^3^ Cooper Medical School of Rowan University Camden New Jersey USA; ^4^ Department of Pathology Cooper University Hospital Camden New Jersey USA; ^5^ Department of Gastroenterology Cooper University Hospital Camden New Jersey USA

**Keywords:** cervical cancer, gastric cancer, metastatic gastric tumor, metastatic tumors

## Abstract

MGTs most commonly arise from malignant melanoma. MGTs from primary cervical cancers are an extremely rare clinical finding with only a limited number of cases reported in the literature to date. We present the case of a 41‐year‐old female who presented with complaints of dysphagia and fatigue with a history of advanced cervical cancer. Endoscopy results revealed an infiltrating metastatic mass, with biopsy findings consistent with squamous gastric carcinoma of metastatic origin. Despite being on parenteral nutrition, poor disease prognosis led to eventually being discharged to hospice.

**Summary**

- Metastatic gastric tumors (MGT) from cervical cancer, though rare, can present with nonspecific symptoms such as dysphagia.
- Early identification is vital for appropriate management, as these tumors often indicate advanced disease and carry a poor prognosis.

## 1 Introduction

Cervical cancer is known for its tendency to infiltrate locally, leading to serious complications including but not limited to ureteral strictures, renal failure due to obstruction, urosepsis, and local bleeding, all of which are highly likely to be regarded as common causes of mortality in these patients [1]. Distant metastasis from primary cervical cancer to the gastric tissue is rare and has only been reported in isolated case reports [2, 3, 4, 5, 6, 7, 8]. Gastric metastatic tumors can arise from various primary cancers however, they predominantly originate from malignant melanoma (23%), breast (15%), and lung (9%) cancers. The stomach is an unusual site for metastasis from cervical cancer with a reported incidence of 0.2%–0.9% [9, 10, 11]. The clinical manifestations of metastatic tumors, such as abdominal pain, nausea, vomiting, and bleeding, are often nonspecific. Herein, we present the case of a 41‐year‐old woman with cervical cancer who developed an MGT secondary to cervical cancer, presenting with new‐onset dysphagia.

## 2 Case History and Examination

A 41‐year‐old woman, previously diagnosed with squamous cell carcinoma of the cervix presented to the emergency department with new‐onset dysphagia and constant mealtime nausea and vomiting for 3 weeks. Her cancer diagnosis was complicated by metastases to the lungs, bladder, and brain. She had completed five cycles of carboplatin, paclitaxel, bevacizumab, and pembrolizumab. Additionally, she had undergone whole‐brain radiation for brain metastases and had ureteral stents placed due to bilateral hydronephrosis from ureteral obstruction. She reported a persistent globus sensation, leading to nausea, occasional vomiting, dysphagia to solids only, odynophagia, decreased appetite, and worsening fatigue. She additionally complained of a cough, which she had had for about 4 weeks.

On arrival at the ED, her blood pressure was 107/77 mmHg, pulse at 93 bpm, temperature at 98°F (36.7°C) via oral measurement, and a respiratory rate of 16 breaths per minute. On physical examination, the patient appeared cachectic and ill‐appearing, requiring 3 L of oxygen via nasal cannula. A lung examination revealed bilateral rhonchi and decreased breath sounds at the bases. The cardiac assessment showed tachycardia with an irregular rate consistent with atrial fibrillation (AFib), with heart sounds S1 and S2 noted. The abdomen was soft and non‐tender, while the extremities showed no edema. Significant muscle wasting and loss of subcutaneous fat, and dry mucous membranes were noted.

## 3 Method (Investigations and Treatment)

Her laboratory workup was evident for anemia with a hemoglobin of 9.1 (normal 12–15.5) and a high alkaline phosphatase (ALP) level (287; normal 20–140). Rest of the workup was unremarkable.

A computed tomography (CT) scan performed on the patient revealed a progressive increase in the left hepatic dome lesion since October 2023, potentially indicating a perfusion defect or infarct. Additionally, new hypodense lesions were observed in both the left and right lobes of the liver compared to prior examinations. There was also evidence of new periportal and gastrohepatic ligament lymphadenopathy, along with a cluster of prominent retroperitoneal lymph nodes, predominantly on the left. Furthermore, findings included splenomegaly and splenic infarction, as well as a trace of fluid in the pelvis.

**FIGURE 1:**
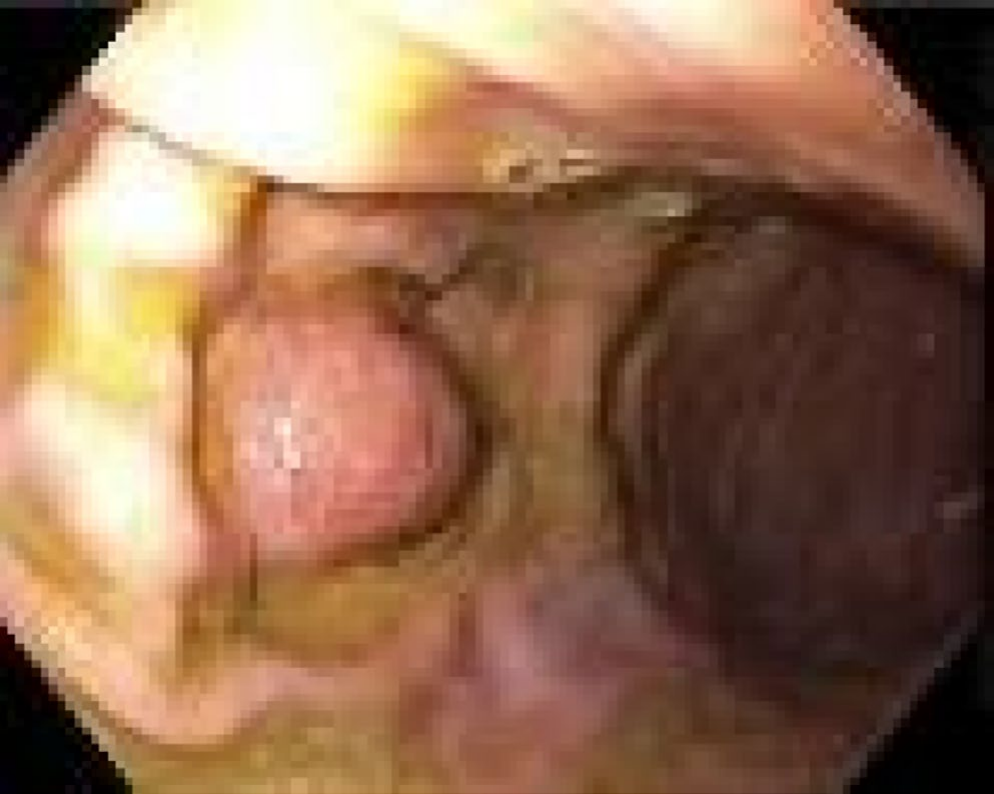
Endoscopic view of the lesion. EGD shows an ulcerating fungating mass at the greater curvature of the stomach.

**FIGURE 2:**
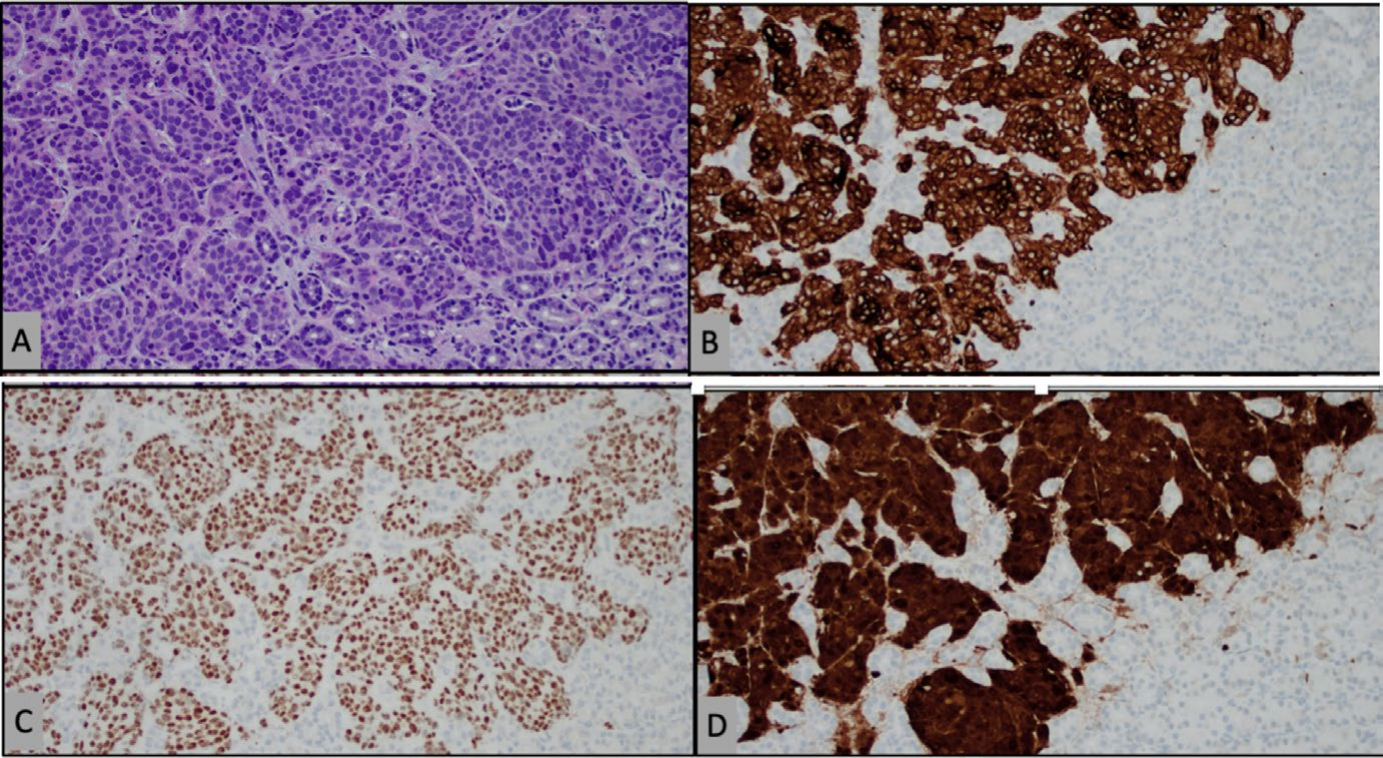
Histological findings of endoscopic biopsy. (A) Hematoxylin and eosin stains show the presence of malignant squamous cells identical to those in cervical cancer, 200× magnification. Immunohistochemistry staining was positive for CK5/6 (B), p40 (C), and p16 (D), consistent with metastatic squamous cell carcinoma of the stomach.

An esophagogastroduodenoscopy (EGD) performed revealed the presence of food residue in the stomach, leading to the premature termination of the procedure. Additionally, findings indicated LA grade A esophagitis. It was recommended that the patient undergo a repeat EGD as an outpatient to further evaluate and manage these findings. Repeat EGD 2 weeks later revealed a medium‐sized, fungating, infiltrative, and ulcerated mass on the greater curvature of the stomach, without associated bleeding or stigmata of recent bleeding (Figure 1). Biopsy results from the EGD showed findings consistent with a malignant squamous cell carcinoma on the greater curvature of the stomach due to metastasis from her known primary cervical cancer. Esophagitis and metastatic gastric cancer are the most likely causes of the dysphagia experienced by this patient. The tumor cells were positive for CK5/6, p40, p16, and CAM5.2 (Figure 2A–D). The ki‐67 proliferation index was up to 60% in tumor cells.

## 4 Conclusion and Results (Outcome and Follow‐Up)

The patient was found to have malignant bilateral pleural effusions, for which she underwent two right thoracenteses and placement of a right PleurX catheter. The cytological analysis confirmed the presence of cervical cancer. An echocardiogram identified a right ventricular echodensity; however, anticoagulation was deemed inappropriate due to the presence of brain metastases. Due to her inability to meet caloric goals, the patient was initiated on partial parenteral nutrition (PPN) for nutritional support. In light of worsening shortness of breath and overall functional decline, she was ultimately discharged to hospice care.

## 5 Discussion

Among cancer patients who develop metastasis leading to secondary cancer, approximately 0.2%–1.7% develop MGT [12]. Although the risk of metastatic disease is low, primary cancers of the lung, breast, and esophagus can metastasize to the stomach, with malignant melanoma having the highest likelihood of primary malignancy [4].

The pathogenesis underlying gastric metastasis is not clearly understood however, it is different for every primary cancer. The four pathways involved in the development of MGTs include peritoneal dissemination, hematogenous dissemination, lymphatic spread, and local tumor invasion [12]. Most gastric metastases involving those from distant organs originate via hematogenous spread. On the contrary, gastric metastases from esophageal carcinoma usually exhibit lymphatic dissemination through the submucosal longitudinal lymphatic network.

A solitary lesion is a more common presentation of MGT than multiple lesions. 40% of solitary lesions in the stomach are located in the upper third around the greater curvature, and 40% are located in the middle third with lesions in the antrum being less common [6, 13, 14]. Endoscopic findings from our case resonated well with existing literature as the MGT was located in the greater curvature of the stomach on EGD.

When gastric tumors are encountered in patients with a known history of primary cervical cancer, it is more likely to be a primary gastric lesion since metastasis to the stomach is rare. This is supported by the clinical presentation of MGT being unspecific and mimicking primary malignancies of the stomach. These symptoms include dysphagia, anorexia, abdominal pain, nausea, vomiting, and dyspepsia but the most common symptom is upper gastrointestinal (GI) bleeding, which presents as anemia and failure to thrive [1, 4]. Our patient presented with profound dysphagia, nausea, vomiting, and weakness which is similar to a typical presentation for MGT.

MGT has a poor prognosis with a median survival period of less than 5 months. Very few cases of MGT qualify for treatment with chemotherapy at the time of diagnosis due to an overall decline in health [6]. Moldovan et al. reported one case of antral stenosis secondary to MGT successfully achieving complete remission after undergoing treatment with adjuvant chemotherapy and radiation [14].

Early diagnosis and intervention remain key in managing MGT through a wide selection of chemotherapeutic and palliative radiation therapies. This report aims to increase the awareness of physicians to enlighten them about the potential of metastatic gastric cancer occurring secondary to malignancies of solid organs.

## Author Contributions

**Fariha Hasan:** conceptualization, data curation, formal analysis, investigation, methodology, project administration, resources, supervision, writing – original draft, writing – review and editing. **Yumna Timsaal:** conceptualization, data curation, formal analysis, investigation, methodology, resources, writing – original draft, writing – review and editing. **Tanay‐Veer Gandhi:** data curation, formal analysis, investigation, methodology, resources, writing – review and editing. **Hadi Shojaei:** methodology, resources, supervision, visualization, writing – review and editing. **Michael Schwartz:** investigation, project administration, resources, supervision, writing – review and editing. **Adib Chaaya:** investigation, project administration, resources, supervision, writing – review and editing.

## Consent

Written informed consent was obtained from the patient to publish this report in accordance with the journal's patient consent policy.

## Conflicts of Interest

The authors declare no conflicts of interest.

## Data Availability

All data generated or analyzed in this study are included in this published article. Additional inquiries can be addressed to the corresponding author.

## References

[1] R. Kanthan , J. L. Senger , D. Diudea , and S. Kanthan , “A Review of Duodenal Metastases From Squamous Cell Carcinoma of the Cervix Presenting as an Upper Gastrointestinal Bleed,” World Journal of Surgical Oncology 9 (2011): 113, 10.1186/1477-7819-9-113. 21958048 PMC3206441

[2] M. Dhanushkodi , S. Krishnan , V. Christopher , and S. Ganesharaja , “Gastric Metastasis From Cervical Cancer,” Indian Journal of Gynecologic Oncology 17 (2019): 24, 10.1007/s40944-019-0268-3.

[3] D. H. Kim , J. H. Kim , Y. C. Lee , et al., “A Case of Metastatic Squamous Cell Carcinomas From the Endocervix to the Stomach Without Local Recurrence,” Korean Journal of Helicobacter and Upper Gastrointestinal Research 13, no. 1 (2013): 55–59.

[4] C. Simões , L. Carrilho‐Ribeiro , and J. Velosa , “Gastric Metastasis From Cervical Carcinoma, Rare Cause of Gastrointestinal Bleeding,” Gastroenterología y Hepatología 42, no. 4 (2019): 249–250, 10.1016/j.gastrohep.2018.04.009. 29866510

[5] A. Singhal , S. Gupta , H. Mohan , and N. Singh , “Gastric and Colonic Metastasis From Cancer Cervix: An Unusual Progression With an Uncommon Cause of Mortality,” South Asian Jornal of Cancer 4, no. 1 (2015): 51–53. 10.4103/2278-330X.149959 PMC4382793 25839029

[6] M. Oriuchi , K. Uno , F. Fujishima , et al., “A Rare Case of Gastric Squamous‐Cell Carcinoma Metastasized From the Cervix,” Clinical Journal of Gastroenterology 13 (2020): 1062–1065, 10.1007/s12328-020-01191-8. 32712841

[7] M. H. Kim , K. A. Kim , Y. K. Chun , J. W. Kim , J. Lee , and C. H. Lee , “Gastric Metastasis From Gastric‐Type Mucinous Adenocarcinoma of Uterine Cervix: A Case Report,” Journal of the Korean Society of Radiology 85, no. 2 (2024): 445–450, 10.3348/jksr.2023.0103. 38617873 PMC11009144

[8] O. V. Topuz and S. Bağbudar , “Cervical Cancer Metastasis to the Stomach Masquerading as Primary Gastric Cancer: A Case Report of a Rare Metastasis Detected via 18F‐FDG PET/CT,” Nuklearmedizin 63, no. 3 (2024): 221–223, 10.1055/a-2224-9673. 38190994

[9] O. Kobayashi , H. Murakami , T. Yoshida , et al., “Clinical Diagnosis of Metastatic Gastric Tumors: Clinicopathologic Findings and Prognosis of Nine Patients in a Single Cancer Center,” World Journal of Surgery 28, no. 6 (2004): 548–551, 10.1007/s00268-004-7216-8. 15366743

[10] P. M. Campoli , F. H. Ejima , D. M. Cardoso , et al., “Metastatic Cancer to the Stomach,” Gastric Cancer 9, no. 1 (2006): 19–25, 10.1007/s10120-005-0352-5. 16557432

[11] K. H. Oda , T. Yamao , D. Saito , et al., “Metastatic Tumors to the Stomach: Analysis of 54 Patients Diagnosed at Endoscopy and 347 Autopsy Cases,” Endoscopy 33, no. 6 (2001): 507–510, 10.1055/s-2001-14960. 11437044

[12] G. H. Kim , J. Y. Ahn , H. Y. Jung , et al., “Clinical and Endoscopic Features of Metastatic Tumors in the Stomach,” Gut Liver 9, no. 5 (2015): 615–622, 10.5009/gnl14032. 25473071 PMC4562778

[13] G. D. De Palma , S. Masone , M. Rega , et al., “Metastatic Tumors to the Stomach: Clinical and Endoscopic Features,” World Journal of Gastroenterology 12, no. 45 (2006): 7326–7328, 10.3748/wjg.v12.i45.7326. 17143949 PMC4087491

[14] B. Moldovan , E. Banu , D. Pocreaţă , et al., “Gastric Metastasis of Cervix Uteri Carcinoma, Rare Cause of Lower Gastric Stenosis,” Chirurgia (Bucur) 107, no. 6 (2012): 816–820. 23294965

